# Polyphenolic Extracts From Green Vegetables as Promoters of Fibroblast Viability and Reducers of Oxidative Stress

**DOI:** 10.1002/fsn3.70230

**Published:** 2025-05-12

**Authors:** Daniela Oprea, Daniel Crisan, Adrian Enache

**Affiliations:** ^1^ National Institute of Materials Physics Magurele Romania; ^2^ Faculty of Physics University of Bucharest Măgurele Romania

**Keywords:** acidified methanolic extracts, DPPH assay, green leafy plant's antioxidants, L929 cells, reactive oxygen species

## Abstract

Over the past decades, extensive research has demonstrated and confirmed the antioxidant potency of polyphenols, which due to these properties, are now widely recognized for their ability to enhance cell viability. This study investigates the antioxidant capacity of seven green leafy vegetables on in vitro mouse (L929) and human (BJ) fibroblast cell lines, aiming to determine if polyphenols could aid the regeneration of connective tissue, which would be strongly beneficial in wound healing and tissue repair, a process where fibroblast thriving is essential. The antioxidant capacity of extracts was investigated using the 2,2‐diphenyl‐1‐picrylhydrazyl (DPPH) assay, UV–Vis spectroscopy, and amperometry, with quercetin serving as a standard. The EC_50_ values for the extracts were equivalent to approximately 5 μM quercetin, derived from a concentration range of 20 to 100 mg of fresh leaves per mL. In vitro investigations revealed that all extracts, except lovage, promoted high viability (over 80%) in both cell cultures, as shown by MTS assay results and fluorescence microscopy. Additionally, Tumor Necrosis Factor (TNF)‐α and Lipopolysaccharide (LPS) were employed as reactive oxygen species (ROS) antagonists, and the Dichloro‐dihydro‐fluorescein diacetate (DCFH‐DA) assay was used to demonstrate the extract's scavenging activity on fibroblasts in vitro. For some extracts, a reduction in oxidative stress compared to the cells basal metabolic conditions was observed.

## Graphical abstract

Selected plant extracts promote cell viability and decrease the effect of oxidative stress.

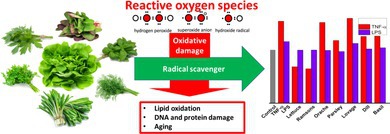

## 1 Introduction

Green leafy vegetables are a rich source of vitamins, minerals, polyphenolic antioxidants, and much more health empowering elements. Polyphenols have been shown to enhance cell viability and proliferation in fibroblast cultures, and by promoting cell growth and survival, polyphenols support the maintenance and regeneration of connective tissues, which can be particularly beneficial for injury recovery and tissue regeneration processes where fibroblast proliferation is essential (Tyszka‐Czochara et al. 2014).

From a nutritional point of view, Table 1, leafy greens embody a low amount of lipids and proteins and are characterized by a low glycemic index, related to their low glucose and low total amount of carbohydrates content, which recommends them for a healthy diet based on a low calorie consumption. On the contrary, the nutritious values of these vegetables are highlighted by a high content of microelements, dietary fibers, and antioxidants (Randhawa et al. 2015). A clear distinction must be made between nutritional and nutritious; while nutritional means “using food to support life” the meaning of nutritious is “eat healthy and take advantages of all nutrients” (Nordquist 2020; Brinkman et al. 2010).

**TABLE 1:** Selected plants and their nutrition values (FoodData Central, n.d.; Rinchen and Singh 2015).

	Binomial name	Nutrition values per 100 g				
		Calories (kcal)	Protein (g)	Lipids (g)	Carbohydrates (g)	Dietary fibers (g)
Lettuce	*Lactuca sativa*	15	1.4	0.2	1.6	1.3
Ramsons	*Allium ursinum*	19	0.9	0.3	2.9	2.2
Orache	*Atriplex hortensis*	26	3.0	3.0	0.0	2.6
Parsley	*Petroselinum crispum*	36	3.0	0.8	2.7	3.3
Lovage	*Levisticum officinale*	42	3.5	0.8	2.0	3.0
Dill	*Anethum graveolens*	43	3.5	1.1	4.9	2.1
Basil	*Ocimum basilicum*	22	3.2	0.6	1.1	1.6

Based on their antioxidant properties, the bioactive molecules of green leafy vegetables are recognized to neutralize free radicals produced by intense physical exercise or by polycyclic aromatic hydrocarbons (PAHs) generated from the digestion of fried, smoked, or grilled meat. During the cooking of meat, especially when using methods like grilling, frying, or smoking, polycyclic aromatic hydrocarbons (PAHs) can be formed (Flores et al. 2019; Bulanda and Janoszka 2022). These PAHs can lead to the production of ROS when metabolized by the body, causing oxidative damage to cells and tissues (Zahed et al. 2023). This damage has been associated with health issues such as inflammation, DNA degradation, and increased risks of cancer and cardiovascular diseases. Additionally, physical exercise, especially strenuous or prolonged workouts, can also trigger ROS production in the body. This occurs due to increased metabolic activity and higher oxygen muscle consumption. Factors like elevated oxygen intake, increased heart rate, and muscle contractions collectively contribute to ROS production during physical activity (Mrakic‐Sposta et al. 2015). While some level of ROS generated during physical exercise can have advantageous training effects, the excessive ROS resulting from sport practice or from consuming grilled, fried, or smoked meat can surpass the body's natural defenses, causing oxidative cellular stress. To manage ROS production during rigorous workouts and meat consumption, moderation in both cases is generally recommended. Additionally, incorporating side dishes rich in polyphenols can help mitigate oxidative stress (Gomes et al. 2012; Duedahl‐Olesen and Ionas 2022; Wang et al. 2019).

According to IUPAC, antioxidant activity deals with the kinetics of a reaction between an antioxidant and the pro‐oxidant or radical it reduces or scavenges, whereas antioxidant capacity measures the thermodynamic conversion efficiency of an oxidant probe upon reaction with an antioxidant. Measuring the antioxidant activity/capacity levels of food is carried out for the meaningful comparison of the antioxidant content of foodstuffs (Apak et al. 2013).

The nature of edible foliage's antioxidant activity/capacity comes either from vitamins and vitamins derivatives, or from their polyphenol component. While vitamin's antioxidant capacity is limited to vitamins C and E or derivatives, the polyphenolic compounds are represented by a very large number of molecules (Chiorcea‐Paquim et al. 2020). The main polyphenolic compounds that can be found in greens belong to the flavonoids and phenolic acids families (DuPont et al. 2000; Justesen and Knuthsen 2001a) and their antioxidant properties are given by resorcinol and/or catechol moieties (Zatloukalová et al. 2013), for example, quercetin and Caffeic acid.

The main characteristic of an antioxidant molecule is given by its ability to neutralize free radicals through electron transfer (Brand‐Williams et al. 1995); a process that employs the oxidation of the antioxidant molecule and the reduction/neutralization of the free radical (Gil and Couto 2013). In fact, electron‐transfer reactions are characteristic features of a variety of fundamental biological processes (Oliveira‐Brett 2017), including metabolism biosynthesis—a process that generates a large number of free radicals (Fang et al. 2002). While the mechanistic complexity of biological electron‐transfer reactions varies considerably from case to case, the underlying principles are the same. Due to its distribution of electrons, the catechol moiety of a flavonoid—which has the ability to transfer two electrons—will be the first one involved in the neutralization of free radicals. However, once the resorcinol moiety is oxidized by the reactive species, its oxidation products will be able to neutralize many more molecules than one catechol moiety (Enache and Oliveira‐Brett 2011).

Usually, the analysis of the plants or fruits antioxidant content begins with the extraction of the antioxidant fraction and is followed by the antioxidant capacity evaluation (Koźmiński and Oliveira‐Brett 2008; Issaad et al. 2017, 2019). Different extraction methods were experimented with and improved over time, and it was proved that the acidified methanolic ultrasound‐assisted method was the most efficient (Koźmiński and Oliveira‐Brett 2008) while the common evaluation methods used for the antioxidant capacity are spectrophotometric and electrochemical (Chiorcea‐Paquim et al. 2020; Mishra et al. 2012; Chen et al. 2013).

In this work, the antioxidant capacity of three green leafy plants (lettuce, ramsons, and orache) and four aromatic plants (parsley, lovage, dill and basil), Table 1, was evaluated using the spectrometric DPPH assay, and the shielding against ROS was tested in vitro using the fibroblast BJ and L929 cell lines.

## 2 Experimental

### 2.1 Materials

The foliage (lettuce, ramsons, orache, parsley, lovage, dill and basil) was purchased from the local market (Bucharest, Romania) at the end of April, and the extracts were prepared within 24 h.

Methanol (Cat. Nr. 34,860), ethanol (Cat. Nr. 51,976) and hydrochloric acid (Cat. Nr. 320,331) for extraction, DPPH (Cat. Nr. D9132) for antioxidant capacity evaluation, and quercetin (Cat. Nr. Q4951) as a standard were purchased from Merck (Germany) and were used without further purification. HPLC CHROMASOLV grade methanol (Cat. Nr. 603–001‐00‐X) was purchased from Honeywell. H_2_O for HPLC was purified using a Merck Millipore Direct‐Q 3 UV water purification system and degassed by sonication. Trifluoroacetic acid (Cat. Nr. A12198) was purchased from Alfa Aesar.

The fibroblast cell lines L929 (Cat Nr. CCL‐1) and BJ (Cat Nr. CRL‐2522) from ATCC (USA), Dulbecco's Modified Eagle Medium (DMEM) (Cat Nr. 302002) from ATCC (USA), Trypsin (Cat Nr.15090046) from ThermoFisher Scientific (USA) and phosphate buffer saline (PBS) (Cat Nr. 70011044) from ThermoFisher Scientific, and Fluorescein Phalloidin (Cat Nr. F432) from ThermoFisher Scientific (USA) were used for in vitro evaluation of antioxidant capacity of plant extracts. Fetal bovine serum (Cat Nr. F7524) from Sigma‐Aldrich, Penicillin–Streptomycin (Cat Nr.15140122), formaldehyde (Cat Nr. 252549) and glutaraldehyde (cat. Nr. 340855) from Sigma‐Aldrich, MTS‐CellTiter 96 AQueous One Solution Cell Proliferation Assay (Cat Nr. G3582) from Promega, Tumor Necrosis Factor (TNF‐alpha) (Cat Nr. 172400000) from ThermoFisher Scientific (USA), and Lipopolysaccharide (LPS) (Cat Nr. 00–4976‐93) were used to assess cell viability and radical scavenging activity.

All necessary solutions were prepared with analytical grade reagents and purified water from a Millipore Milli‐Q system (conductivity ≤ 0.1 μS/cm).

### 2.2 Instrumentation

#### 2.2.1 Spectrophotometry

DPPH, H_2_DCFDA, and MTS were performed using a FLUOstar Omega multi‐mode microplate reader (BMG LABTECH, Germany).

#### 2.2.2 Fluorescence Microscopy

The fluorescence microscopy images were obtained using a Leica DM6B upright fluorescence microscope (Leica Microsystems CMS GmbH) equipped with a Leica CTR6 LED (electronic box containing the power supply for the electronics and the lamps) and a Leica EL6000 external light source for fluorescence excitation. The samples were imaged using a 40× objective (0.65 NA, 0.36 mm WD and correction ring) from Leica, the appropriate filter cube (excitation filter 480/50 nm, dichroic mirror 505–510 nm and emission filter 527/30 nm) and the 4.2 MP sCMOS Leica DFC9000 monochrome fluorescence camera.

#### 2.2.3 Electrochemical Measurements

Electrochemical measurements were recorded at the RS Electrochemical Detector of the Thermo Fisher Scientific Dionex UltiMate 3000 UPHLC^+^, used for automatic injection of samples. The samples were analyzed at 25°C using an isocratic mobile phase (5% MeOH with 0.1% TFA, 95% H_2_O with 0.1% TFA) and a flow rate of 0.5 mL/min using a dummy column to capture all the antioxidant species at the same retention time with the solvent front. The samples were analyzed at 300, 700, and 900 mV respectively.

### 2.3 Methods

#### 2.3.1 Plant Extract

For each plant, 25 g of leaves were washed with ethanol and water and crushed. Extraction of the polyphenolic compounds was performed in three steps, using a mixture of methanol, ethanol, and hydrochloric acid, 8:1.9:0.1(v:v:v), as extraction solvent. First, the crushed leaves were mixed with 10 mL of extraction solvent, placed in a microwave oven at 450 W for 30 s, protected from the light with aluminium foil, immersed in an ultrasound bath, and subjected to ultrasound treatment at 25°C and a constant frequency of 35 kHz for 15 min. After 1 h of incubation in the dark at room temperature (22°C to 24°C) the samples were centrifuged at 3500 rpm in a TX‐150 rotor (model SL 8R centrifuge Thermo Fisher Scientific, Germany) for 20 min, and the supernatant was collected in the laboratory flask and stored at +4°C. In the second step, the remaining solids of each plant were mixed with 10 mL of extraction solvent, subjected to ultrasound treatment for 30 min, centrifuged, and the extracts were collected and mixed with those obtained in the first step. This step was repeated once more. For each plant, the extracts obtained in all three steps were mixed and filtered using a 45 μm bottle‐top sterile filter and a vacuum pump.

#### 2.3.2 Spectrophotometric Evaluation of Antioxidant Capacity by DPPH Assay

Absorbance spectrum of each plant extract was recorded using the FLUOstar Omega multi‐mode microplate reader and a 96‐well plate. The experimental conditions were: the total volume of 130 μL/well (corresponding to a path length of 1 cm), a scan speed of 400 nm/min, and the sampling interval of 1 nm. The DPPH stock solutions were prepared before each experiment, and a concentration of 100 μM was used. In order to optimize the response linearity and establish the antioxidant capacity in the adequate linear range, 12 different concentrations have been assayed for each plant extract. It should be mentioned that, between sample preparation and measurement, there was a Δt of 300 s.

The DPPH radical scavenging activity (RSA), %RSA = (1‐A_sample_/A_DPPH_) x 100, was calculated using the DPPH maximum absorbance measured at 516 nm. The antioxidant capacity was expressed as the EC_50_ value, defined as the amount of antioxidant necessary to decrease the DPPH absorbance, measured at 516 nm, by 50% of the initial absorbance. All experiments were done in triplicate (*n* = 3) at room temperature.

#### 2.3.3 Electrochemical Index

The total polyphenolics, as Electrochemical Index (EI) and the high antioxidant power of phenolics were obtained using the electrochemical analysis and a modified screening protocol proposed originally by Blasco (Blasco et al. 2005). Briefly, the extracts were injected into the electrochemical flow cell, using the automation of an HPLC system, and the oxidation currents were measured at 300 mV for the evaluation of high antioxidant power of phenols and at +900 mV for the electrochemical index.

#### 2.3.4 Cells Cultivation

The L929 murine and BJ human fibroblast cell lines were grown in Dulbecco's Modified Eagle Medium (DMEM) culture medium supplemented with 4.5 g/L glucose, 2 mM l‐glutamine, 10% fetal bovine serum, and penicillin (100 U/mL), streptomycin (100 μg/mL), under controlled conditions (95% humidity, 5% CO_2_, 37°C). Sub‐cultivation was done in cell culture flasks (T 25) and when cells reached pre‐confluence ~80%, the cells were detached using a trypsin solution of 0.25% concentration. Unless otherwise specified, L929 cells were counted and seeded in 96 well plates at a density of 10,000 cells/well, and BJ cells were seeded at a density of 4000 cells/well.

#### 2.3.5 Cell Viability and Intracellular ROS Assay

The MTS cell viability kit is a complete, optimized assay that generates a consistent colorimetric detection of viable mammalian cells. The effect of plant extracts, at different experimental conditions (see Section 2.3.4), on cell viability was determined after 24 h incubation by measuring the absorbance at 490 nm using the microplate reader. All samples were run in triplicate in the same assay.

The total ROS generated by cells upon 24 h incubation with the oxidative stress agonists was evaluated using the H_2_DCFDA assay. The assay is based on the conversion of non‐fluorescent 2',7'‐dichlorofluorescin diacetate (DCFH‐DA) to highly fluorescent 2',7'‐dichlorofluorescein (DCF) in the presence of ROS. 2',7'‐dichlorodihydrofluorescein diacetate enters the cells, where intracellular ROS is present, and cleaves the acetate groups, releasing the fluorescent molecule 2',7'‐dichlorofluorescein. The fluorescence was measured at 520 nm, and all samples were run in triplicate. For the evaluation of total ROS, seeding was performed in a 96 well plate at 7000 cells/well density for murine cells and at 4000 cells/well density for human cells and incubated for 24 h under specific condition (95% humidity, 5% CO_2_, 37°C). Next, the cell cultures were subjected to the plant extracts for 24 h and treated with oxidative stress agonists, TNF‐alpha (50 ng/mL) and LPS (50 ng/mL) for 24 h. Similar treatment was performed on the human cell line for ROS evaluation, BJ cells being seeded in a 96 well plate at 4000 cells/well density and incubated for 24 h under specific conditions, subjected to the plant extracts for another 24 h, and finally treated with the stress agonists, TNF‐alpha (50 ng/mL) and LPS (50 ng/mL) for 40 min. For the MTS assay, after 24 h incubation, quercetin and plant extracts at EC_50_ concentrations were added to the cell plates and incubated for another 24 h. The statistical analysis was conducted using a one‐way ANOVA, followed by a Tukey post hoc test, with *p* < 0.05 considered statistically significant.

## 3 Results

The antioxidant capacity, based on DPPH radical and Electro chemical Index (EI) assays, as well as the in vitro effect on cell cultures, of several edible foliage's extracts was investigated using UV–Vis, fluorescence spectroscopy, microscopy, and amperometry.

### 3.1 Evaluation of the Antioxidant Capacity by DPPH Assay

The DPPH assay is a widely used method to evaluate the antioxidant capacity of compounds, particularly plant extracts and natural products. DPPH is a stable free radical compound with a deep purple color in its radical form. When an antioxidant substance is added to a DPPH solution, it reacts with the DPPH radical by donating a hydrogen atom or an electron. This reaction results in the reduction of the DPPH radical, causing a color change from purple to yellow, which can be measured spectrophotometrically.

The spectrophotometric evaluation of the antioxidant capacity was performed by measuring the absorption of DPPH at 520 nm and correlating the absorbance maximum decrease in the presence of extracts with the antioxidant capacity. The half maximum effective concentration (EC_50_) of each extract and quercetin as standard was calculated as the concentration value inducing a 50% decrease of DPPH radical maximum absorbance, that is, the neutralization of half the amount of radical. This spectrophotometric method allows not only the comparison of antioxidant capacity within different extracts but also expresses the capacity as standard equivalent.

**FIGURE 1:**
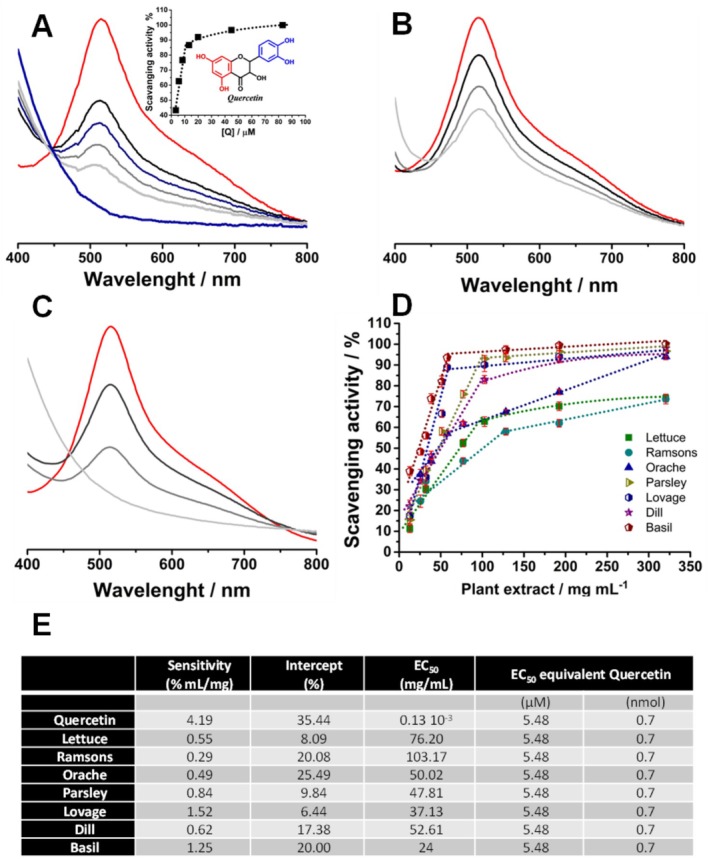
(A) Normalized spectra obtained for (A) 100 μM DPPH (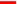) before and after different additions of Quercetin and the scavenging activity (insert), (B) Ramsons and (C) Basil extracts: (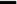) 12.82, (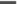) 25.64 and (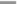) 192.30 mg/mL; (D) the scavenging activity of extracts and (E) the analytical parameters obtained for extracts.

For all experiments, the interaction time was 5 min and it was observed that with increasing quercetin concentration, from 3 to 83 μM, the decrease and disappearance of the maximum absorbance of DPPH takes place, Figure 1A. The decrease of DPPH radical maximum absorbance can be expressed as radical scavenging activity, Figure 1A‐insert, and from the linear dependence of the scavenging activity with the quercetin concentration, an EC_50_ = 5.48 μM for quercetin was calculated, close to the theoretical value and experimental reported values (Chen et al. 2013).

Similar to quercetin, the plant extracts presented the same behavior, i.e., the decrease of maximum absorbance of DPPH with the increase of concentration. However, the decrease varied from extract to extract, Figure 1B,C, and plotting the scavenger activity vs. concentration of the extract, Figure 1D, the EC_50_ values were calculated, Figure 1E.

The lowest EC_50_ values, i.e., the highest antioxidant capacity, were obtained for the aromatic plants basil and lovage while the highest value was found for ramson. Nevertheless, the EC_50_ values were between 20 and 100 mg/mL, meaning that less than 100 mg of fresh leaves has the same antioxidant power as 5.48 μM quercetin, Figure 1E.

### 3.2 Electrochemical Index

Electrochemical methods are very suitable techniques, compatible with the evaluation and quantification of the antioxidant power of different systems, and over time several methods were developed, the most common being Electrochemical Index (EI) (Blasco et al. 2005). The principle of this method is based on the quantification of the electrochemical oxidation current of phenolic groups, i.e., catechol, phenol, and resorcinol, at high potential; the working potential should be above the oxidation potential of resorcinol. At the oxidation potential of phenol, the intermediate antioxidant phenolics power is obtained, whereas at low oxidation potential, where catechol moieties oxidation takes place, the high antioxidant phenolics power can be estimated.

While some derivatives of this method use the ratio sum of the current and the oxidation potential (Lino et al. 2014), in samples containing more redox species or when a chemical species exhibits multiple peaks, the integrated area under the oxidation peak is considered a more comprehensive estimation of the total amount of exchanged electrons (Chevion et al. 2000; Schilder et al. 2020; Haque et al. 2021).

**FIGURE 2:**
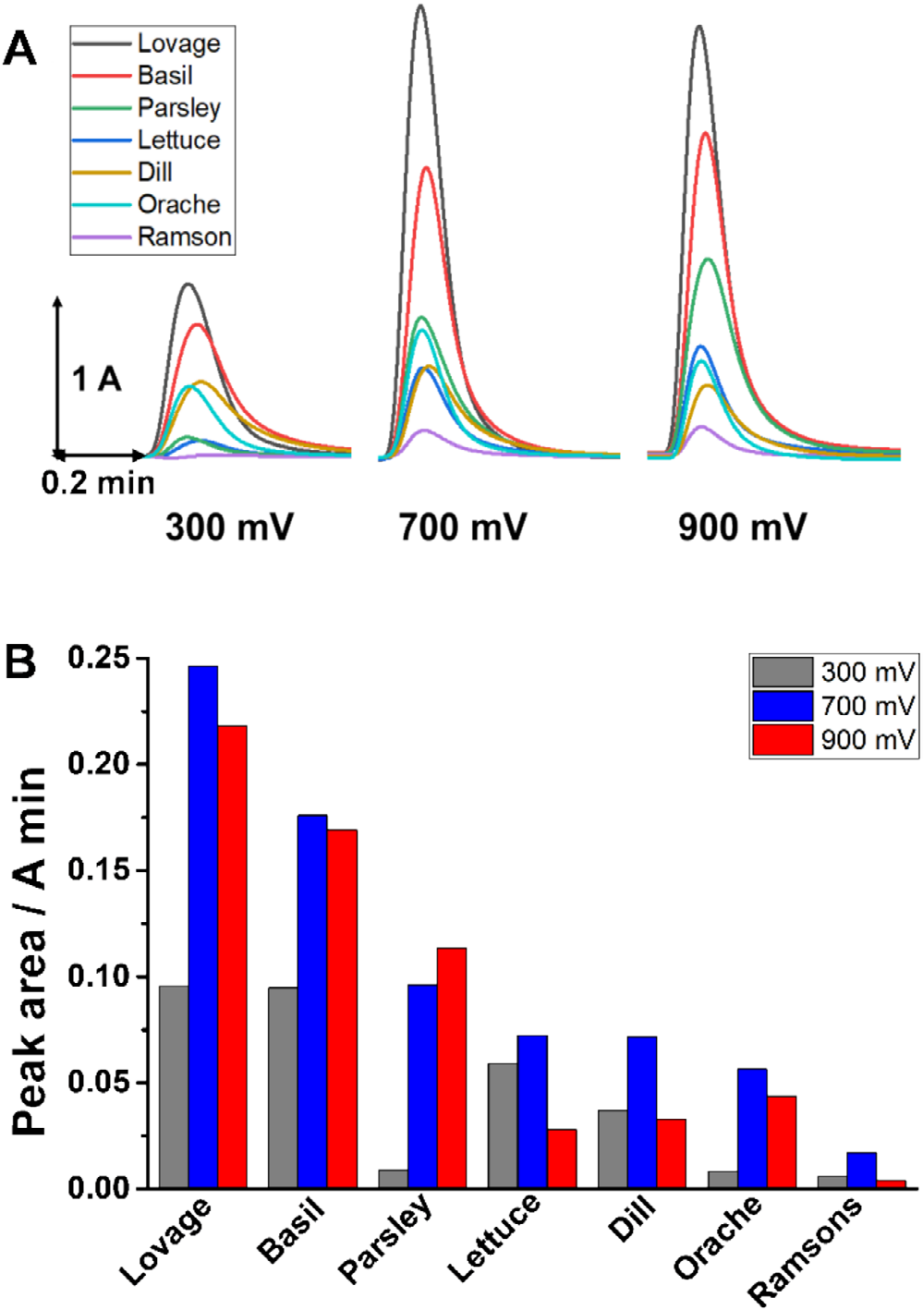
(A) Chronoamperograms obtained for plant extracts at different working potentials, and (B) the corresponding peak area.

Using an electrochemical detector assisted by the injection system of a HPLC equipment, the oxidation peaks of all extracts were obtained at 300, 700, and 900 mV, and the EI was estimated. Thus, the antioxidant power of the extracts can be classified into three categories where lovage and basil are the most powerful, followed by parsley, lettuce, dill, and orache, while the lowest values were obtained for ramsons, Figure 2. One should mention that for all the extracts the measured charge at 700 and 900 mV presented similar values, a common phenomenon occurring in complex matrix.

### 3.3 In Vitro Evaluation

**FIGURE 3:**
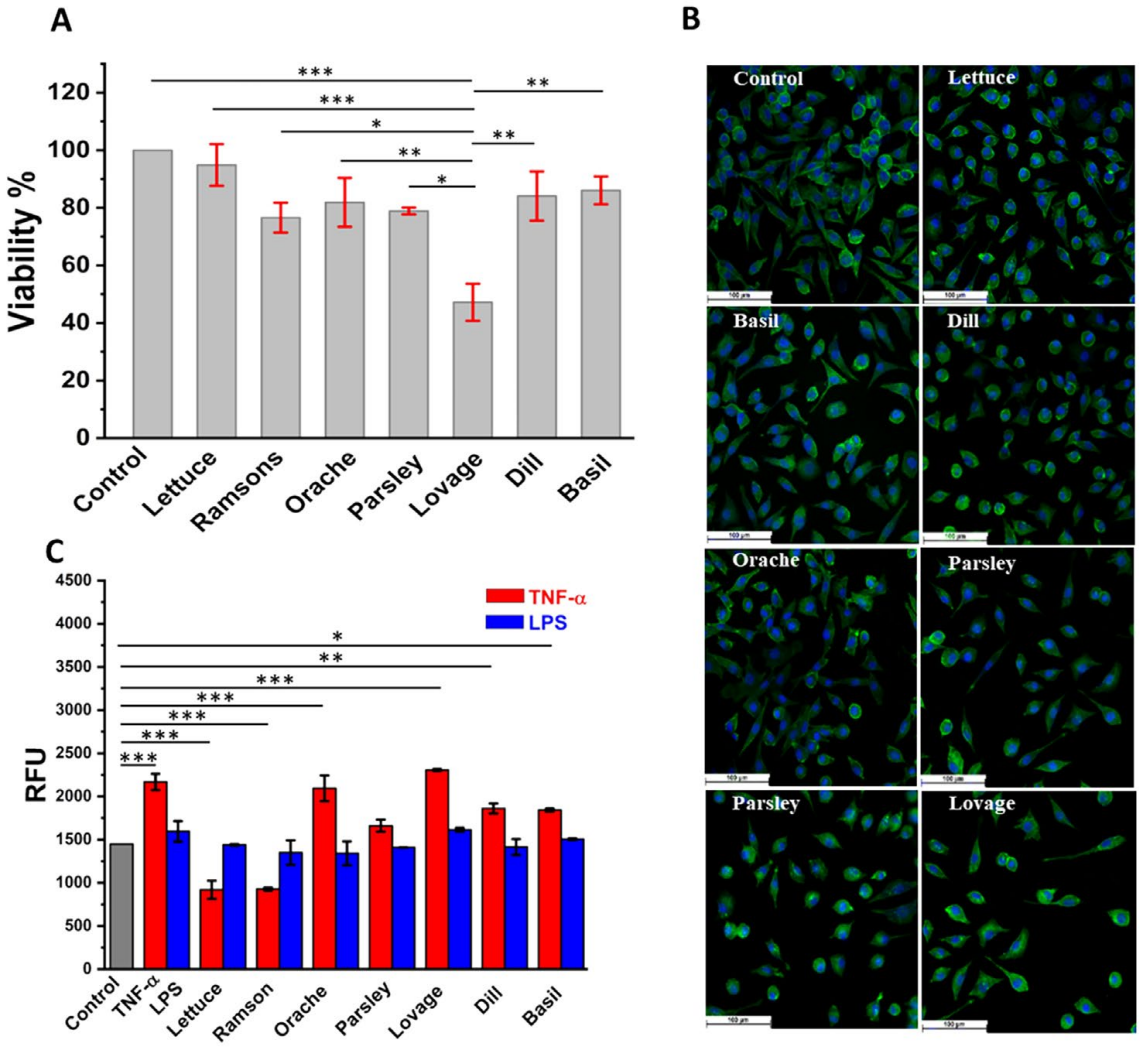
(A) Cell viability obtained for L929 culture incubated with plant extracts (B) Fluorescent images of L929 cell line treated with plat extracts and (C) ROS generated by cell culture incubated with TNF‐α and LPS after 24 h treatment with plant extracts; ***p < 0.001, **p < 0.01, *p < 0.05.

Since the EC_50_ allows the comparison of antioxidant capacity within different extracts related to a standard equivalent, that is, quercetin, the MTS cell viability test was performed to evaluate the biocompatibility of the phenolic plant extracts over BJ and L929 fibroblasts at EC_50_ concentration, Figure 3A.

Cell viability refers to the number of healthy and physiologically functional cells enclosed in a biological working sample, and it measures the integrity and proper functionality of cells in a cell population. A high viability indicates that a majority of cells from a sample are alive and function properly and to the contrary, low viability suggests that a high percentage of dead, dysfunctional cells are present in the studied population. Tetrazolium salt assays such as MTS (3‐(4,5‐dimethylthiazol‐2‐yl)‐5‐(3‐carboxymethoxyphenyl)‐2‐(4‐sulfophenyl)‐2H‐tetrazolium) are extensively used to assess cell viability. MTS assay measures the metabolic activity of cells, which is an indicator of cell viability. In an MTS assay, MTS is reduced by the mitochondrial dehydrogenases (active in viable cells), resulting in the formation of a colored formazan product. The intensity of formazan product is proportional to the number of viable and metabolically active cells of the evaluated culture.

**FIGURE 4:**
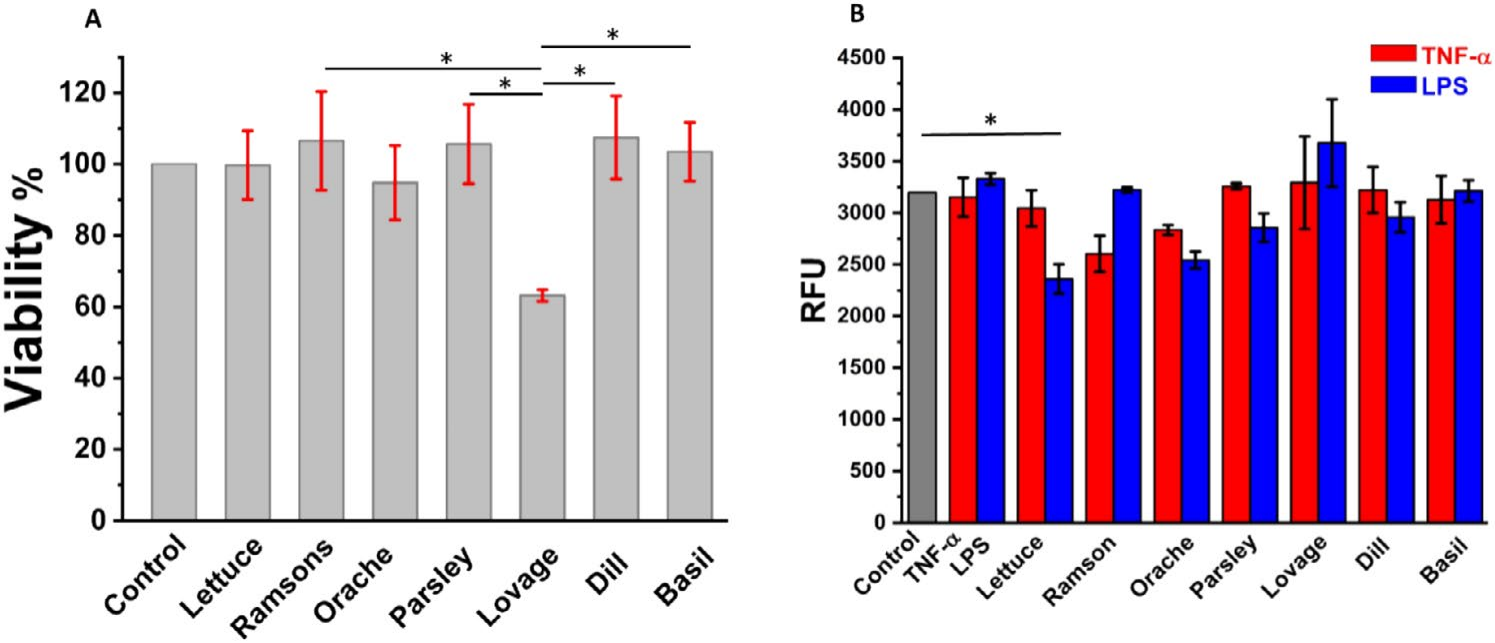
(A) BJ cell viability incubated with plant extracts; (B) ROS generated by BJ cell culture incubated with TNF‐α and LPS after 24 h treatment with plant extracts; *p < 0.05.

The MTS assay was used to analyze the viability of fibroblastic cell lines (L929) and (BJ) after their interaction with the phenolic plant extracts of lettuce, ramsons, orache, parsley, lovage, dill, and basil, at the EC_50_ concentration determined by the DPPH assay. Significant results were obtained for lovage, lettuce, basil, and dill, while the lovage extract showed a certain level of toxicity, reducing the viability of cell cultures by 50% when compared to the control. The viability of the L929 cells treated with lettuce, basil, and dill showed over 90% viability in regard to the control, and ramsons, orache, and parsley expressed over 80% viability. Six of the analyzed extracts, with the exception of lovage, manifested over 90% viability in regard to the control in the case of the BJ cell line, all of which suggests a high degree of non‐toxicity for most of the extracts, Figures 3A and 4A.

Based on the electrochemical index and the cell viability results, the extracts were subjected to fluorescence microscopy in order to complementarily investigate their interaction with L929 fibroblast cells. Lettuce, basil, dill, orache, parsley, and ramson extracts interaction with L929 cells presented a viability and morphology similar to control, and no harmful effect was observed. The interaction between lovage extract and cells presented a high toxicity toward the cell culture, characterized by the decrease in cell number compared to the control.

Apart from obvious aspects of viability, few extensive remarks can be marked in regard to the morphological characteristics of the treated cells. Mature fibroblastic cells have a well‐defined spindle‐shaped or elongated morphology and the cell body is relatively larger than the nucleus. The nucleus of mature fibroblastic cells is typically single, centrally located, and relatively small in proportion to the cell size. It often appears elongated and can have a smooth, rounded shape. In contrast, young fibroblasts can have a more rounded or less elongated morphology compared to mature fibroblastic cells. They may appear smaller and less spindle‐shaped. The nucleus in young fibroblasts may be larger and less elongated than in mature fibrocytes, and it may have a more irregular shape. Morphological characteristics of mature fibroblasts enable them to perform their essential functions in maintaining tissue structure, repairing injuries, and synthesizing the extracellular matrix, components necessary for tissue integrity. Young fibroblasts, in change, being morphologically undeveloped, have a very low physiological activity (Chong et al. 2019; Cooper 2019). It can be observed in Figure 3B that most of the cells exhibit morphological characteristics of active mature fibroblasts, except for those incubated with lovage extract that show a high number of inactive cells in rapport with its total number of cells and compared to the control.

Based on the fluorescence assay—H2DCFDA, the level of ROS present in the cells was spectrophotometrically measured and associated with the oxidative stress induced by the cytokine TNF‐α and by the outer Gram‐negative bacteria membrane's molecule—LPS agonists that triggered a robust inflammatory reaction over L929 fibroblastic cells. The outcome of this test revealed that, TNF‐α induced a 30% and LPS 10% more oxidative stress in L929 cells compared to the basal metabolism and, exceptionally, lettuce and ramson phenolic extracts demonstrated the capability to reduce oxidative stress by more than 50% in response to TNF‐α, decreasing ROS by 25% under basal metabolism, Figure 3C. Parsley, dill, and basil had 20% effectiveness in neutralizing free radicals induced by TNF‐α, while orache and lovage, in change, had no effect in reducing cellular stress produced by TNF‐α. The amount of free radicals induced by LPS was lowered to the level of basal metabolism by lettuce, ramson, orache, parsley, dill, and basil; only lovage was 10% over the basal metabolism of cell ROS level, Figure 3C.

Considering the metabolic differences between murine and human cells, viability (MTS) and fluorescence (H2DCFDA) assays were performed on the human fibroblast BJ cell line. However, given that both TNF‐α and LPS treatments can induce ROS production fairly rapidly within 15–30 min post‐treatment (Hsu and Wen 2002), the incubation time with ROS antagonists was reduced to 40 min. Similar to L929 fibroblasts, the viability test performed on the BJ cell line after 24 h of incubation with plant extracts at the EC_50_ concentration, determined by the DPPH assay, showed that lovage extract decreased cell viability by 40%. In contrast, some extracts, such as ramsons, parsley, and dill, resulted in viability values slightly above the control, and lettuce and orache presented similar values to control, Figure 4A.

The outcome of chemically induced ROS in BJ cells revealed that neither TNF‐α nor LPS induced significantly higher oxidative stress compared to basal metabolism. This suggests that BJ cells possess a relatively stable redox balance under these conditions or have efficient endogenous antioxidant mechanisms that counteract ROS production. However, as shown in Figure 4B, post‐treatment with certain plant extracts, prior to ROS antagonists, led to a reduction in ROS levels induced by either TNF‐α or LPS, bringing them below control levels. This indicates that the antioxidant bioactive compounds of these extracts are capable of modulating oxidative stress responses in BJ cells. Similar to the results obtained for L929 fibroblasts, treatment with lovage extract resulted in a notable increase in total ROS production. Considering the results of the viability assays, this effect may be explained by the complex interplay between lovage's high antioxidant activity and its potential pro‐oxidant behavior under specific conditions. Lovage is known to contain significant amounts of flavonoids and polyphenols, particularly quercetin—an antioxidant molecule with well‐documented chemotherapeutic properties (Szparaga et al. 2023). Quercetin has been shown to exert both antioxidant and pro‐oxidant effects depending on its concentration, cellular environment, and interaction with other biomolecules. Notably, quercetin is present in large amounts in lovage, as reported in Phenol‐Explorer database (Justesen and Knuthsen 2001b). The observed increase in ROS production following lovage treatment might be due to quercetin's ability to act as a redox‐active compound, which can paradoxically promote ROS generation under certain conditions, ultimately influencing cell viability.

## 4 Discussion

More than 15 years ago, in 2006, the European Food Safety Authority (EFSA) elaborated a “Scientific Opinion on the substantiation of health claims related to various food(s)/food constituent(s) and protection of cells from premature aging, antioxidant activity, antioxidant content and antioxidant properties, protection of DNA and protection of proteins and lipids from oxidative damage” (Panel and Nda 2023), in order to sustain the “Regulation (EC) No 1924/2006 of the European parliament and of the council, of 20 December 2006, on nutrition and health claims made on food” especially directed to food labelling (Zicari et al. 2007).

The Scientific Opinion was directed to the use of the terms “antioxidant activity” and “antioxidant properties”, in relation to food constituents. It was also addressed the association of the mentioned terms with cells premature aging prevention, antioxidant activity, antioxidant content, and protection of DNA, proteins, and lipids from oxidative damage. The target population was assumed to be the general population.

The Panel has found there are neither in vivo nor clinical studies that evidence the fact that antioxidant activity/content and/or antioxidant properties have a beneficial physiological effect and based on that time available data, concluded that no cause and effect relationship could have been established between the consumption of the evaluated food constituents and any positive physiological effect associated with antioxidant activity, antioxidant content, antioxidant properties, or the protection of cells from premature aging.

As for the effects pertaining to the protection of cells and biomolecules such as lipids, proteins, or DNA from oxidative damage, including damage induced by UV radiation over the intended target population, the Panel considered the possibility that there may indeed be a beneficial physiological effect. Even so, after further evaluation based on available data, it was noticed that the evidence presented from animal and in vitro studies was insufficient to predict whether the food(s)/food constituent(s) would have an effect on protecting body cells and molecules, such as DNA, proteins, and lipids, from oxidative damage in humans.

**FIGURE 5:**
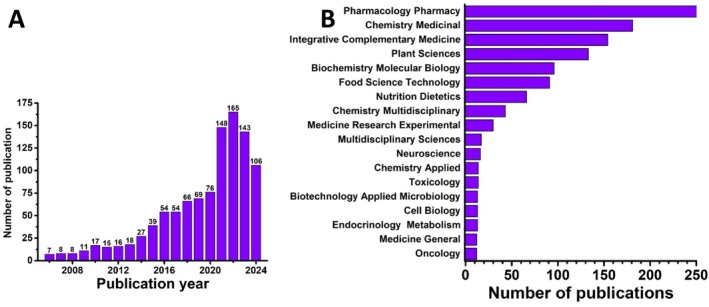
(A) Number of publications and (B) Article distribution per years.

It should be mentioned that the results returned by the Web of Science database for 1996–2006, for the key words “antioxidants”, “plant extracts,” “clinical studies,” and “clinical trials” return 20 articles, while the results from 2006 to January 2024, returns 1.047 results, of which the review articles represent a considerable number, Figure 5.

Although, in the last 5 years, there is an increased effort of investigation and a high amount of evidence about the beneficial effects of plant antioxidants, the recommendations of EFSA about precaution on labeling should still stand. Many plant antioxidants have therapeutic effects, but the abuse can lead to undesirable effects (Vafadar et al. 2020).

In this study, it is shown that the lovage extract, at the same antioxidant capacity as lettuce, reduced the viability in murine and human fibroblast cell lines by 50%, a fact that can be explained through the high content of quercetin, a biomolecule used as cancer therapy to dismantle malignant cells. Overall, the green plants under investigation in this article present high antioxidant activity, low cytotoxicity, and beneficial properties for cells under oxidative stress induced by bacterial LPS or by the cytokine TNF‐α.

Taking into consideration the EC_50_ values for each plant extract calculated using the DPPH assay, the DPPH concentration of 100 μM and the working volume of 130 μL, a salad mixture consisting of lettuce, ramson, orache, and aromatic herbs such as parsley, basil, and dill possesses the capability to neutralize a total amount of 150 μmol of free radicals, Table 2.

**TABLE 2:** Representation of plant's radical scavenging activity.

A	B	C	D	E	F
	EC 50 (mg/mL)	in 130 μL		Mix salad (g)	Reactive potential (μmol)
		Plant (mg)	Scavenger value of DPPH (nmol)		
Lettuce	76.2	9.9	6.5	100	65.6
Ramson	103.17	13.4	6.5	30	14.5
Orache	50.02	6.5	6.5	50	50
Parsley	47.81	6.2	6.5	5	5.2
Basil	24	3.12	6.5	5	10.4
Dill	52.61	6.8	6.5	5	4.7
Total					150.5

This amount of free radicals may be equivalent to 40 min of progressive arm‐ergometer training, a pre‐exercise performed by master swimmers before swimming training sessions. (Mrakic‐Sposta et al. 2015). In fact, lowering the basal ROS level by reducing ROS production is the first (a) from the redox ABC (Li 2013) and it can be achieved either through regular exercise or/and by having a healthy, balanced diet. Furthermore, a side dish salad such as the one proposed in Table 2 (column E) may also have the potential to counteract the production of free radicals resulting from polycyclic aromatic hydrocarbons (PAH) after a meal that involves grilled meat digestive metabolization (Wang et al. 2019; Sampaio et al. 2021).

## 5 Conclusions

Using the ultrasound acidified methanol method, the polyphenolic content of several leafy edible greens was extracted and the radical scavenger activity, as well as the total antioxidant capacity, were evaluated by the means of UV–Vis spectroscopy and electrochemistry.

The spectrophotometric evaluation of the antioxidant capacity was performed by DPPH assay, measuring the absorption at 520 nm, and the half maximum effective concentration (EC_50_) of each extract was calculated as the concentration value which produced a 50% decrease of DPPH radical maximum absorbance, that is, neutralization of half amount of radical. Also, the antioxidant capacity of the extracts was evaluated using amperometry at fixed potential; 0.3 V (vs. Ag/AgCl) was chosen for the evaluation of high antioxidant content and 0.7 V for the estimation of total antioxidant fraction. The amperometric results were in good agreement with the EC_50_ values; the lowest EC_50_ values were obtained for the highest current oxidation peaks.

Based on these results, the plant extracts, at the concentration of EC_50_ value, were assessed in L929 and BJ fibroblast cell cultures, measuring viability and the protective effect of plant extracts against oxidative stress induced by LPS and TNF‐α. Significant results have been achieved in the case of lettuce and ramson, which compared to the cellular basal metabolism decreased with ~ 25% the level of free radicals produced by murine cells and with 20% respectively 15%, the level of free radicals produced by human cells after they have been stressed with LPS and TNF‐α. Finally, a salad of ~ 200 g with a ~ 150 μmol antioxidant capacity was proposed using 6 of the plants that were analyzed.

## Author Contributions

**Daniela Oprea:** data curation (equal), formal analysis (equal), investigation (equal), validation (equal), visualization (equal), writing – original draft (equal), writing – review and editing (equal). **Daniel Crisan:** formal analysis (equal), investigation (equal), validation (equal), writing – original draft (equal). **Adrian Enache:** conceptualization (lead), data curation (lead), formal analysis (lead), funding acquisition (lead), investigation (lead), methodology (lead), project administration (lead), resources (lead), supervision (lead), validation (equal), visualization (equal), writing – original draft (equal), writing – review and editing (equal).

## Conflicts of Interest

The authors declare no conflicts of interest.

## Data Availability

The authors have nothing to report.
